# Eosinophil-Driven vs. Eosinophil-Associated Severe Asthma: Practical Implications for Target Treatment

**DOI:** 10.3390/ijms26041729

**Published:** 2025-02-18

**Authors:** Valentina D’Aiuto, Ilaria Mormile, Francescopaolo Granata, Antonio Romano, Francesca Della Casa, Gabriele Mignogna, Amato de Paulis, Francesca Wanda Rossi

**Affiliations:** 1 Department of Translational Medical Sciences, University of Naples Federico II, 80131 Naples, Italy; valedaiuto@tiscali.it (V.D.); frapagra@hotmail.com (F.G.); francesca.dellacasa4@gmail.com (F.D.C.); depaulis@unina.it (A.d.P.); francescawanda.rossi@unina.it (F.W.R.); 2 Head and Neck Section, Department of Neurosciences, Reproductive and Odontostomatological Sciences, University of Naples Federico II, 80131 Naples, Italy; romano.antonio1972@gmail.com; 3 Post-Graduate Program in Clinical Immunology and Allergy, University of Naples Federico II, 80131 Naples, Italy; gabrymig@gmail.com; 4 Center for Basic and Clinical Immunology Research (CISI) University of Naples Federico II, 80131 Naples, Italy; 5 World Allergy Organization (WAO) Center of Excellence, 80131 Naples, Italy

**Keywords:** severe asthma, type 2 inflammation, eosinophils, biological therapies, atopy biomarkers, personalized medicine

## Abstract

Severe asthma (SA) is a chronic inflammatory condition affecting approximately 10% of asthmatic patients, and eosinophils are considered key pathogenetic actors in a significant number of patients. Biological therapies have been demonstrated to improve asthma control by decreasing exacerbation rates and reducing the use of oral corticosteroids. In this context, phenotyping and endotyping patients with SA is essential for selecting the most effective therapeutic approach. For this purpose, biomarkers such as IgE, absolute blood eosinophil count, and fractional exhaled nitric oxide (FeNO) are crucial in defining a patient’s inflammatory profile. Their integration provides a framework for classifying asthma into T2-high, T2-mild, or T2-low categories, guiding personalized treatment strategies. By incorporating multiple biomarkers into a unified model, it is possible to better stratify patients and optimize biologic therapy selection, paving the way for improved outcomes in SA management. This review aims to evaluate the role of phenotyping and endotyping SA patients, with particular attention to the impact of eosinophilic inflammation and combinatory biomarkers on decision-making processes for the selection of biological therapies.

## 1. Introduction

Asthma is a chronic respiratory disease characterized by persistent lung inflammation affecting up to 18% of the global population [1]. Severe asthma (SA) is defined by the European Respiratory Society/American Thoracic Society task force as an asthma subtype, which requires high doses of inhaled corticosteroids (ICS) plus a second controller drug and/or oral corticosteroids (OCS) to achieve control, or which remains uncontrolled despite this therapy [2]. Uncontrolled asthma is defined by the presence of at least one of the following characteristics: persistently poor symptom control, two or more exacerbations requiring bursts of systemic corticosteroids in the preceding year, at least one serious exacerbation requiring hospitalization in the previous year, or chronic airflow limitation with FEV1 < 80% predicted and an FEV1/FVC ratio below the lower limit of normal [3]. According to the European Network for Understanding the Mechanisms of Severe Asthma (ENFUMOSA), approximately 10% of asthmatic patients develop SA [1]. SA is associated with significant morbidity and mortality [4]. Furthermore, the quality of life of patients with SA is significantly impaired by drug side effects, particularly OCS [4]. In this view, biological therapies have emerged as steroid-sparing tools to achieve disease control in patients with SA in the long term [5,6]. The United States Food and Drug Administration (FDA) and the European Medicines Agency (EMA) have approved several monoclonal antibodies including anti-IgE (omalizumab), anti-interleukin (IL)-5 (reslizumab and mepolizumab), anti-IL5Ralpha (benralizumab), anti-IL4/IL13(dupilumab), and more recently, anti-thymic stromal lymphopoietin (TSLP) (tezepelumab) [7]. These drugs target critical molecular pathways in asthma pathogenesis, modulating inflammation and airway hyperresponsiveness, significantly improving symptoms and lung function, and reducing exacerbations and the use of OCS [8,9,10,11,12,13,14,15,16]. However, not all patients experience sufficient clinical benefit from biologics, often requiring switching to an alternative drug [17]. Indeed, the effectiveness of biologic therapy greatly depends on appropriate patient selection, which requires a detailed analysis of patient characteristics, medical history, and the evaluation of multiple biomarkers [18]. Advances in understanding the pathophysiological mechanisms of asthma have enabled the distinction between phenotypes (observable characteristics) and endotypes (underlying mechanisms) [19]. Endo-phenotyping patients with SA is critical for selecting a priori the optimal biologic drug from the wide currently available pharmacological armamentarium [20]. In this review we aim to evaluate the role of phenotyping and endotyping patients with SA for optimal management of this condition, particularly focusing on the impact of eosinophilic inflammation and the role of combinatory biomarkers on decision-making processes for the selection of the appropriate biological therapy.

## 2. Asthma Clinical Phenotype and Endotype

The definition of clinical phenotypes in SA is based on a combination of clinical characteristics, including the time of onset (early vs late), lung function, treatment response, concomitance of atopic diseases, comorbidities (e.g., obesity, chronic rhinosinusitis with or without nasal polyps, gastroesophageal reflux disease, atopic dermatitis, urticaria, eosinophilic esophagitis, and aspirin-exacerbated respiratory disease), and conditions resulting from steroid use [21,22,23,24]. Based on the predominant immunological pathways guiding the inflammatory processes and determining the phenotypes, asthma can be further classified into two major inflammatory endotypes: type 2 (T2)-high and non-T2-high (T2-low) [25,26]. The T2-high endotype is usually associated with some degree of eosinophilic airway inflammation and a variable allergic or non-allergic background [27]. Furthermore, the Global Initiative for Asthma (GINA) and the European Academy of Allergy and Clinical Immunology (EAACI) Biologicals Guidelines identify two subsets of disease: allergic asthma and eosinophilic asthma [28,29]. Allergic asthma, by definition, implies atopy and often also eosinophilia, even though the latter is not a constant association [30]. Symptoms are correlated with serum total IgE (tIgE) levels from 30 to 1300 IU/mL and triggered by exposure to perennial aeroallergens (i.e., house dust mites, animal dander, and molds) in sensitized patients [31]. This atopic reactivity is driven by the helper T lymphocyte subset 2 (Th2), which depends on the interaction between specific T-cell receptors (TcRs) and allergen fragments presented by dendritic cells [32]. Accordingly, tIgE, specific IgE (sIgE), and skin prick tests (SPT) are used as biomarkers for assessing allergic status [33,34]. Eosinophilic asthma (EA) is characterized by elevated eosinophil levels in the airways, peripheral blood, and sputum and is defined by at least one of the following criteria: blood eosinophils ≥ 150 cells/mL, fractional exhaled nitric (FeNO) ≥ 20 ppb, or sputum eosinophils ≥ 1% [28,35,36]. EA can be either allergic or non-allergic [37]. Allergic eosinophilic asthma is typically Th2 mediated (Th2-high), while non-allergic eosinophilic asthma primarily involves innate lymphoid cells type 2 (ILC-2) [37]. This highlights that, in the pathogenesis of SA, allergic conditions and eosinophilia can coexist or can be unrelated [32,38].

The T2-low endotype, on the other hand, is characterized by: (i) low levels of T2 biomarkers, (ii) neutrophilic or paucigranulocytic inflammation (non-allergic/non-eosinophilic response), and (iii) activation of Th1 and/or Th17 cells [22,25].

In this background, the evaluation of T2 biomarkers helps in endophenotyping patients and categorizing their asthma profiles along the spectrum from T2-high to T2-low [39]. In our view, integrating the patient’s allergic condition (tIgE and specific sensitization) with eosinophilic status (blood or sputum eosinophils or FeNO) allows us to identify four distinct categories of SA patients: (i) allergic/eosinophilic (T2 high): patients in this category are both allergic and eosinophilic, indicating a strong T2-driven inflammatory response; (ii) non-allergic/non-eosinophilic (T2 low): these patients are neither allergic nor eosinophilic, suggesting a minimal or absent T2-driven inflammatory profile; (iii) non-allergic/eosinophilic: This group includes patients who are not allergic but show a predominant eosinophilic inflammation mainly driven by ILC2 activation; (iv) allergic/non-eosinophilic: This group includes patients who are allergic but not eosinophilic reflecting a moderate level of T2 inflammation with prevalent T helper 2 activation. We referred to these last two groups as T2 mild to emphasize the partial activation of T2 inflammation [25] (Table 1).

## 3. Eosinophils in Severe Asthma

Eosinophils are granulocytic white blood cells derived from hematopoietic stem cells in the bone marrow [40]. IL-5 is an essential cytokine for their differentiation, maturation, and release into the bloodstream [40]. Additional cytokines, such as IL-3 and granulocyte-macrophage colony-stimulating factor (GM-CSF), further modulate their activation and sustain their survival in tissues [41]. In the bloodstream, eosinophils have a short half-life of approximately 8–12 h. However, once they migrate into tissues, their lifespan can extend significantly, lasting from days to weeks, particularly when sustained by a local inflammatory cytokine milieu [42,43]. Under normal conditions, eosinophils are predominantly located in mucosal tissues, such as the gastrointestinal tract, where they play a role in immune surveillance [44].

Eosinophils contain intracellular granules packed with cytotoxic and pro-inflammatory proteins that are critical in immune defense and inflammation. Key components of these granules include eosinophil cationic protein (ECP), major basic protein (MBP), eosinophil peroxidase (EPO), and eosinophil-derived neurotoxin (EDN) [45]. In addition, eosinophils produce lipid mediators such as leukotrienes (e.g., LTC4, LTD4, LTE4), which amplify inflammation by promoting bronchoconstriction, vascular permeability, and mucus secretion. They also secrete cytokines, including TGF-β, IL-1β, IL-6, and TNF-α, which perpetuate inflammation and contribute to airway remodeling processes, such as fibrosis and smooth muscle hypertrophy [46]. In asthma (particularly T2-high severe asthma), eosinophils are actively recruited to airway tissues in response to chemokines upregulated by IL-4 and IL-13 such as eotaxin-1 (CCL11), eotaxin-2 (CCL24), and eotaxin-3 (CCL26), which binds to CCR3 receptors on eosinophils [37]. Once in the airway tissue, eosinophils degranulate, releasing their cytotoxic proteins and other mediators, which significantly contribute to the pathophysiology of the disease [47]. The harmful effect of eosinophilic activation in asthma is well-documented. Studies have demonstrated a strong correlation between eosinophilia, poor asthma control, and increased exacerbation rates [48,49,50]. This evidence has contributed to identifying eosinophils as a therapeutic target, leading to the development of biologics such as mepolizumab and benralizumab [51]. These therapies have shown significant benefits in patients with severe eosinophilic asthma, including improved lung function and reduced exacerbations [52].

## 4. Identification of Eosinophilic Asthma and Combinatorial Use of Biomarkers

Eosinophils, FeNO, and IgE (tIgE and sIgE) are considered the most relevant biomarkers of the T2 inflammatory pathway [30,36]. These biomarkers can coexist or present independently, gradually varying between the T2 high and T2 low settings [39,53]. Although the presence of a dominant biomarker is highly desirable, most patients with SA often do not exhibit a single dominant biomarker [18,54]. For example, data from the international severe asthma registry showed that stratifying patients with SA by eosinophil count, tIgE, and FeNO, more than 80% of patients with a given biomarker presented the concomitance of at least another additional biomarker with a variable frequency rate of association [54]. Accordingly, Guida et al. [18] reported that 70% of patients with SA tested positive for at least two biomarkers. Moreover, the recent multicentric registry-based cohort study by Porsbjerg et al. evaluating the pre- and post-biologic change in biomarker concentrations (i.e., BEC, FeNO, and IgE) in patients with SA show that these markers were all elevated at the pre-biologic assessment [55].

Eosinophil count in induced sputum is considered the gold standard as a biomarker of T2 inflammation, with variable cut-off [18,56,57,58,59,60]. However, it is a complex and time-consuming procedure that requires trained personnel, the availability of resuscitation facilities, and a laboratory with specific experience [61,62,63]. Therefore, absolute eosinophil count (AEC) from the peripheral blood and FeNO are often used in clinical practice as substitute markers due to their easier measurement [25]. For AEC, blood eosinophil thresholds of ≥150 and ≥300 cells/μL are commonly applied to identify patients with eosinophilic asthma [64], although these values fall within the normal range of 500 cells/μL [65]. Therefore, a truly reliable threshold value of AEC for diagnosing eosinophilic asthma has not yet been established [32]. In addition, it is clear that multiple eosinophil measurements over time can better reflect patients’ eosinophilic status, accounting for both interindividual variability and intraindividual fluctuation [64]. Indeed, even though the optimal number of AEC measurements has yet to be established, the last GINA update (available at https://ginasthma.org/2024-report/, accessed on 20 January 2024) recommends repeating measurement of AEC up to 3x, at least 1–2 weeks after OCS or on lowest possible OCS dose. In line with this suggestion, in a recent study, our research team collected N. 4 AEC determinations over a 12-month period from patients with bronchial asthma, allergic rhinitis, or chronic rhinosinusitis with (CRSwNP) or without nasal polyposis, all suffering from seasonal allergies to parietaria and grasses [66]. The Mean AEC (MAEC) was then calculated. We found that an MAEC of 400 cells/μL was the most appropriate threshold for distinguishing eosinophilic from non-eosinophilic patients in our cohort [66]. Indeed, the use of MAEC reduces the risk of intra-individual fluctuations derived from extrinsic and intrinsic factors and prevents the underestimation or overestimation of the eosinophilic status potentially resulting from a single AEC measurement [66]. In addition, as proposed by Toledo-Pons et al. [67], the availability of multiple AEC measurements allows to calculate the eosinophil variability index (EVI) as (Eosmax − Eosmin / Eosmax) × 100%. An EVI ≥ 50% has been shown to be a better predictor for any hospital episode than any absolute count value [67].

Bronchial FeNO, the other non-invasive biomarker of T2 and eosinophilic airway inflammation [36,68], shows variability in cutoff values across different clinical guidelines [69]. Values <25 ppb (or <20 ppb for children) are generally considered low or normal, while values >50 ppb (or >35 ppb for children) strongly suggest type 2 inflammation and eosinophilic involvement [36,70]. Values between 25 and 50 ppb (or 20–35 ppb for children) are considered elevated but should be interpreted in the context of the patient’s clinical history and other available biomarkers [71].

Similarly to eosinophil counts and FeNO, allergy biomarkers also exhibit a gradual distribution. Elevated tIgE is generally defined as serum levels exceeding 100 kU/L [72], although various other reference ranges have been proposed [73,74,75]. For example, the current guidelines for omalizumab use in allergic asthma recommend its indication in cases of perennial allergen sensitization combined with tIgE levels >30 or >76 kU/L, depending on the country [76]. By contrast, patients with extremely high serum tIgE levels (>1300 kU/L) are typically excluded from eligibility [76].

**Figure 1:**
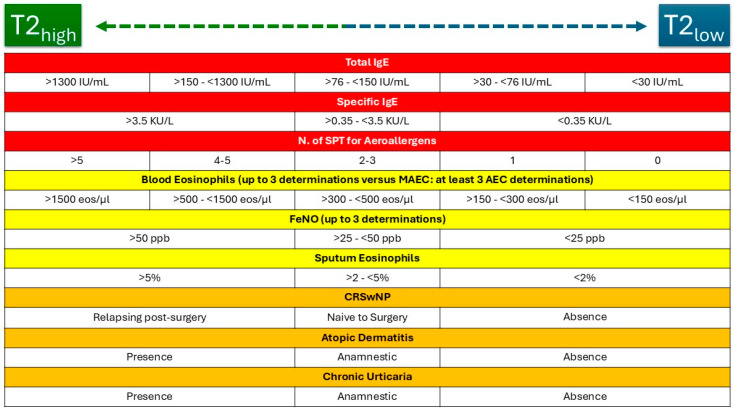
Proposed grading of T2 biomarkers and comorbidities routinely used for endo-phenotyping severe asthma patients. CRSwNP, chronic rhinosinusitis with nasal polyposis; SPT, skin prick tests; FeNO, fractional exhaled nitric oxide. Color legend: red, allergic asthma; yellow, eosinophilic asthma; orange, comorbidities.

Regarding sIgE, sensitization is defined as the presence of at least one allergen-specific IgE level ≥ 0.35 kUa/L for a given allergen [77]. Higher values or the number of allergen-specific sensitizations are often associated with a pronounced T2-high profile [75]. As shown in Figure 1, T2 biomarkers can be distributed according to the cutoffs used by the scientific community along a gradient that progresses from low T2 to high T2 settings [75]. In addition, allergic comorbidities such as CRSwNP, atopic dermatitis, and chronic urticaria often align with a high T2 profile [78]. On the contrary, their absence is more characteristic of a T2-low profile [78].

Grading of biomarkers and their combinations offers a refined approach to patient stratification [28]. To benefit from the grading of blood eosinophil count in patients with severe asthma, we propose to avoid the use of a single AEC and suggest the use of a mean absolute eosinophil count (MAEC) calculated from at least three AECs. Accordingly, the currently used thresholds (i.e., >150 cells/μL or >300 cells/μL) to identify patients with eosinophilic asthma should not be used with a single AEC but rather with MAEC as stated above. In addition, in the attempt to set an additional threshold, we propose 500 cells/µL, which is currently used to identify patients with blood eosinophilia [65]. Furthermore, integrating MAEC thresholds and FeNO values could enhance the characterization of the patient’s endophenotype, with a focus on understanding the contribution of eosinophils to the underlying inflammatory mechanism. For this purpose, we propose a classification system based on the integration of MAEC (calculated from at least three AEC determinations) and FeNO values (up to three determinations), dividing SA into four groups (Table 2):

1. Non-Eosinophilic Asthma is defined by both MAEC and FeNO values below the thresholds that, according to the literature, indicate the absence of eosinophilic inflammation;
2. Low-Eosinophilic Asthma is defined by the presence of MAEC < 150 eos/µL and FeNO > 25 ppb or MAEC ≥ 150 to <300 eos/µL combined with FeNO < 25 ppb;
3. Eosinophil-Associated Asthma is characterized by MAEC ≥ 150 to <300 eos/µL combined with FeNO > 25 ppb or MAEC ≥ 300 to <500 eos/µL combined with FeNO < 25 ppb;
4. Eosinophil-Driven Asthma is defined by MAEC ≥ 300 to <500 eos/µL combined with FeNO > 25 ppb or MAEC ≥ 500 eos/µL combined with any FeNO.

**Table 2:** Proposed classification of severe eosinophilic asthma based on grading of biomarkers. Mean absolute eosinophil count (MAEC) was calculated from at least three absolute eosinophil count (AEC), and fractional exhaled nitric (FeNO) values considering up to three determinations. This classification applies only to patients not on maintenance therapy with oral corticosteroids. NR, not relevant.

Non-Eosinophilic Asthma	
MAEC < 150 cell/µL + FeNO < 25 ppb	
**Low-Eosinophilic Asthma**	
Variant #1 MAEC < 150 eos/µL + FeNO > 25 ppb	Variant #2 MAEC ≥ 150–<300 eos/µL + FeNO < 25 ppb
**Eosinophil-Associated Asthma**	
Variant #1 MAEC ≥ 150–<300 eos/µL + FeNO > 25 ppb	Variant #2 MAEC ≥ 300–<500 eos/µL + FeNO < 25 ppb
**Eosinophil-Driven Asthma**	
Variant #1 MAEC ≥ 300–<500 eos/µL + FeNO > 25 ppb	Variant #2 MAEC ≥ 500 eos/µL + FeNO = NR

It should be noted that evidence to support this proposed classification is currently lacking; therefore, it should be considered an eminent-based recommendation rather than evidence-based. In any case, the categorization into eosinophil-associated and eosinophil-driven asthma may highlight two distinct inflammatory settings: in the former, the presence of eosinophils could represent an epiphenomenon, not necessarily playing a causal role in the underlying inflammatory process of the disease; by contrast, in eosinophil-driven asthma, eosinophils represent the key pathological determinant of the disease process [25,79].

## 5. Biologics Available for Severe Asthma

The FDA has currently approved six biologics for the treatment of severe T2 asthma (i.e., omalizumab, mepolizumab, benralizumab, reslizumab, and tezepelumab). Omalizumab is a recombinant humanized IgG1 monoclonal antibody that targets and binds free IgE, interrupting the IgE-mediated inflammatory cascade [80]. It is indicated for moderate-to-severe persistent asthma in patients who exhibit baseline total serum IgE levels between 30 and 1300 IU/mL and sensitization to at least one perennial aeroallergen confirmed by either a SPT or sIgE [81]. In addition, omalizumab has been approved as an add-on maintenance treatment for CRSwNP in adults with inadequate response to nasal corticosteroids [82] and for chronic spontaneous urticaria [83]. The presence of a perennial allergic sensitization is a predictor of treatment response [83], suggesting the usefulness of omalizumab in patients with a clear type 2 allergic asthma profile in which IgE-mediated mechanisms play a significant role.

Mepolizumab, benralizumab, and reslizumab target eosinophilic inflammation and are recommended as add-on treatments for patients with severe, uncontrolled asthma exhibiting an eosinophilic phenotype [6]. Mepolizumab is a fully humanized monoclonal antibody that binds to IL-5, inhibiting its role as a key cytokine involved in the development, activation, and survival of eosinophils [84,85]. Besides severe eosinophilic asthma, mepolizumab is also approved for the treatment of CRSwNP, hypereosinophilic syndrome, and eosinophilic granulomatosis with polyangiitis [24,86,87]. In clinical trials, mepolizumab has been shown to reduce exacerbation rates, improve lung function, decrease OCS use, and provide clinically significant improvements in patients with a baseline blood eosinophil count (BEC) ≥ 150 cells/mL [11,84].

Similarly to mepolizumab, reslizumab inhibits IL-5 activity by blocking the binding of IL-5 to the receptor expressed on the eosinophils cell surface [12,88]. Its intravenous administration has demonstrated significant improvements in lung function, asthma exacerbations, and QoL. In particular, a reduction of exacerbation rate and improvement in lung function was found in patients with eosinophils ≥400 cells/µL [28]. Currently, reslizumab is approved only as an add-on therapy for SA. A prospective observational study (NCT03369574) aimed at monitoring CRS symptoms in asthma patients undergoing treatment with reslizumab, evaluating the potential benefit of CRS symptoms, but the study has withdrawn.

Benralizumab is a humanized monoclonal antibody targeting the IL-5Ra approved for uncontrolled severe eosinophilic asthma that induces rapid and almost complete eosinophil depletion through enhanced antibody-dependent cell-mediated cytotoxicity [89,90]. It offers clinical benefits for patients with elevated BEC, a history of frequent exacerbations, poor lung function, OCS use, CRSwNP, and adult-onset asthma [91]. In particular, the greatest benefits were observed in patients with eosinophil counts exceeding 300 cells/μL [13]. Both mepolizumab and benralizumab have been shown to be effective regardless of baseline FeNO levels (data about the potential predictive role of FeNO in SA patients are not reported in preclinical studies and phase 2–3 RCTs of reslizumab) [92]. This aligns with the fact that both therapies target IL-5, which directly contributes to eosinophilic inflammation, whereas FeNO is more strongly associated with IL-13-induced inflammation [93].

These data taken together would suggest the choice of mepolizumab, reslizumab, and benralizumab for patients with SA and high eosinophilia, which appears to play a more dominant role than an allergy in the inflammatory pathway [94,95]. Dupilumab is a human IgG4 monoclonal antibody that binds IL-4Rα and inhibits signaling of both IL-4 and IL-13. It is approved for moderate to severe persistent asthma in patients with allergic asthma and is poorly controlled with inhaled corticosteroids [96]. Other approved indications are CRSwNP, atopic dermatitis, prurigo nodularis, and eosinophilic esophagitis [97,98,99]. Reduction in asthma exacerbations, improvement in lung function, and OCS reduction was greater in patients with BEC > 300 eos/μL or FeNO > 25 ppb [100,101], and efficacy of dupilumab was greater in patients with > 1 exacerbation during the 12 months before study initiation as well as in patients with late-onset asthma [102]. Based on its features, dupilumab is particularly indicated in profiles characterized by atopy and elevated FeNO, aligning with its targeted action on IL-4 and IL-13 [103]. Several case reports and post-marketing surveillance have described hypereosinophilia during dupilumab therapy, which may occur in up to 14% of patients, even with severe consequences [104,105,106,107]; therefore, caution is advised when starting therapy with dupilumab in patients with hypereosinophilia (>1500 eos/μL).

Tezepelumab is a human monoclonal antibody that binds to TSLP [108], an epithelial-derived cytokine released from the airway epithelium in response to various environmental triggers and implicated in the T2 inflammatory response, inflammation, and airway hyperreactivity [16,109,110]. Despite better outcomes in patients with higher baseline eosinophils count, tezepelumab has shown clinical efficacy in both T2 high and T2 low asthma, demonstrating good outcomes in patients with BEC < 150 eos/μL and FeNO levels < 25 ppb [110,111,112,113]. The presence of sensitization to perennial allergens does not influence the therapeutic response [112]. Tezepelumab appears particularly suitable for patients with T2-low or mixed inflammation, where no single type 2 biomarker is clearly dominant [114]. Currently, tezepelumab has not been approved for other indications [115].

## 6. Implication of Eosinophil Grading in the Choice of Biologic Therapy

The broad availability of monoclonal antibodies for SA paves the way for a personalized treatment approach. However, several patients present overlapping allergic and eosinophilic features, making them eligible for multiple biologic drugs [116]. The choice of the most suitable biologic should be guided by the predominant biomarker [113], although, as discussed before, several patients exhibit a gradient of multiple biomarkers [117]. As a result, biological therapy is often started not relying on a defined algorithm but based on the individual clinician’s choice.

**Figure 2:**
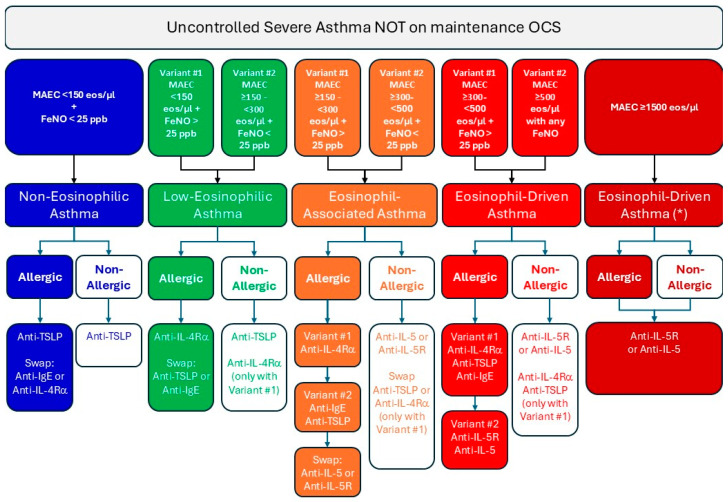
Proposed algorithm for stratifying and tailoring the treatment of patients with varying degrees of eosinophilic and allergic status. OCS, oral corticosteroids. * In these cases, the possibility of hypereosinophilic syndrome (HES) or eosinophilic granulomatosis with polyangiitis (EGPA) should be ruled out.

By combining eosinophilic status (defined through MAEC and FeNO values) with the presence or absence of allergy, we propose a clinical management model aimed at stratifying and tailoring the treatment of patients with varying degrees of eosinophilia and allergy (Figure 2). It should be noted that the algorithm in Figure 2 would be just an example of how a logical approach can be used to interpret the endophenotyping of patients with severe asthma before starting biologic therapy.

In patients with “non-eosinophilic asthma”, characterized by MAEC < 150 eos/µL, FeNO < 25 ppb, and without allergy, tezepelumab emerges as a strong option, given its effectiveness in targeting non-T2 inflammation [118]. However, if the patient is allergic, omalizumab or dupilumab could serve as an alternative [119,120].

In “low-eosinophilic asthma”, the presentation varies slightly. This group includes patients with MAEC < 150 eos/µL and FeNO levels > 25 ppb or patients with MAEC between 150 and 300 eos/µL and FeNO < 25 ppb. For allergic individuals, dupilumab may be the first choice for the former scenario [121], considering tezepelumab or omalizumab [119] as alternative therapies. On the other hand, non-allergic patients in this group could benefit from tezepelumab or dupilumab, depending on their FeNO levels [121,122].

Moving to eosinophil-associated asthma, patients exhibit a more prominent eosinophilic component. Those with MAEC between 150 and 300 eos/µL and FeNO above 25 ppb, or MAEC between 300 and 500 eos/µL and FeNO below 25 ppb, fall into this category. Allergic patients with higher FeNO levels may respond well to dupilumab [121], while those with lower FeNO levels might be better suited for omalizumab [123] or tezepelumab [124]. In non-allergic cases, anti-IL-5 therapies, such as mepolizumab, reslizumab, or benralizumab, are preferred [125], with tezepelumab or dupilumab considered as secondary options in specific contexts [121,126,127].

Finally, eosinophil-driven asthma reflects a condition where eosinophils may act as the primary pathological driver. Patients with MAEC levels between 300 and 500 eos/µL and FeNO levels above 25 ppb, or those with MAEC exceeding 500 eos/µL regardless of FeNO, belong to this group. For allergic individuals, dupilumab [100], omalizumab [128], or tezepelumab [124] may be effective in cases with elevated FeNO, while benralizumab or mepolizumab or reslizumab may be preferred for more severe eosinophilia [84,129,130]. Non-allergic patients, particularly those with very high eosinophil counts, are probably best managed with benralizumab or mepolizumab, or reslizumab [131,132,133] as first-line therapy, with tezepelumab [112], or dupilumab [134] should be considered as a second opportunity. For patients presenting with severe eosinophilia (MAEC > 1500 eos/µL), benralizumab, mepolizumab, or reslizumab remains the primary therapeutic choice [135,136]. Furthermore, in these cases, the possibility of HES or EGPA should be ruled out [137,138,139].

This practical approach emphasizes the importance of considering both eosinophilic and allergic biomarkers, leading to a personalized treatment plan focused on the specific inflammatory mechanism driving each patient’s asthma.

Another factor that should always be considered when selecting therapy for SA is the presence of comorbidities [140]. Indeed, certain comorbid conditions may represent themselves as a specific indication for a particular biologic, further sharpening the choice [141]. For instance, CRSwNP affects over 30% of individuals with SA [142], and dupilumab, omalizumab, and mepolizumab are approved for this condition independently of their indications for asthma [143]. Benralizumab has also been shown to be highly effective in patients with SA and concomitant CRSwNP [91]. Other examples are the concomitance of atopic dermatitis, eosinophilic esophagitis, and prurigo nodularis, which can benefit from dupilumab administration [144]. Similarly, the presence of chronic urticaria, for which omalizumab is indicated, or conditions like HES and EGPA, where mepolizumab is effective, should influence therapeutic decisions [113]. Thus, identifying comorbidities is another critical component for evaluating patients with SA significantly influencing the choice of biologic therapy.

In order to address the unmet needs of patients affected by SA, several biologic and small-molecule therapeutics are in various stages of development, including anti−IL-33 agents (astegolimab and itepekimab) [145], anti−IL-13 [146], chemoattractant receptor−homologous molecule expressed on T-helper type 2 cells [147] or prostaglandin D2 antagonists [148], anti-GATA3 DNAzyme [149], Toll-like receptor 9 agonists [150] and oral small-molecule drugs JAK inhibitors [151].

## 7. Conclusions

SA is a heterogeneous and complex condition that requires a personalized approach to be appropriately managed. Advances in understanding the pathophysiology of this condition have highlighted the importance of endophenotyping patients. In particular, when evaluating eosinophilic status, it should be considered that in some cases, activated eosinophils are the main effector cells and play a major role in the pathogenesis of SA; in other cases, their presence represents an epiphenomenon due to T2 inflammation. Future studies are expected to discriminate between eosinophil-driven asthma and eosinophil-associated asthma. Meanwhile, the integration of multiple biomarkers, including MAEC, FeNO, and IgE levels, may offer a refined framework for patient classification, enabling tailored therapeutic strategies. Additionally, comorbidities such as CRSwNP, atopic dermatitis, and eosinophilic esophagitis provide additional context for therapy selection, as some biologics address both SA and these associated conditions.

## Figures and Tables

**Table 1:** Stratification of SA patients based on endotype and multiple biomarkers.

Endotype		Allergic Biomarkers	Eosinophilic Biomarkers
**Allergic/eosinophilic**	**T2 High**	+	+
**Non-allergic/eosinophilic**	**T2 Mild**	−	+
**Allergic/non-eosinophilic**	**T2 Mild**	+	−
**Non-allergic/non-eosinophilic**	**T2 Low**	−	−
